# Retrospective intrafraction motion verification using triggered kilovoltage images during bone metastases stereotactic body radiotherapy

**DOI:** 10.1016/j.phro.2026.101054

**Published:** 2026-08-03

**Authors:** Quentin Techer, Noé Grandgirard, Saturnin Sandjong, Vincent Marchesi, Paul Retif

**Affiliations:** a Medical Physics Unit, CHR Metz-Thionville, Metz, France; b Medical Physics Department, Institut de cancérologie de Lorraine, 54519, Vandœuvre-Lès-Nancy, France; c Université de Lorraine, CNRS, CRAN, F-54000 Nancy, France

**Keywords:** Bone metastases, SBRT, Intrafraction motion, Triggered kilovoltage imaging, Quality assurance

## Abstract

**Background and purpose:**

Triggered kilovoltage images acquired during radiotherapy delivery remain underexploited for quantitative intrafraction motion analysis. This study aimed to develop and evaluate a retrospective workflow for intrafraction motion verification using triggered kilovoltage images during bone metastases stereotactic body radiotherapy.

**Materials and methods:**

Triggered kilovoltage images and corresponding treatment-planning data were retrospectively extracted from 49 patients treated in two radiotherapy centers. For each triggered image, a digitally reconstructed radiograph was generated from planning computed tomography dataset. After preprocessing, rigid in-plane registration between triggered images and digitally reconstructed radiographs was performed. The workflow was evaluated using phantom experiments with known physical displacements and patient datasets with simulated shifts, then applied to 8717 triggered images acquired during 183 treatment fractions.

**Results:**

Correct registration was obtained for 7105 images (81.5%), while 1612 images (18.5%) were classified as aberrant and excluded from motion analysis. After exclusion, the mean displacement vector magnitude was 1.2 ± 0.8 mm. Overall, 85% of measurements showed displacements ≤2 mm and 98% showed displacements ≤3 mm. Sustained displacement events exceeding 2 mm over three consecutive images were observed in 405 of 7105 images (5.7%) and in 46 of 159 analyzable fractions (28.9%). The mean computation time per image registration was 1699 ± 504 ms.

**Conclusions:**

Triggered kilovoltage images contain quantitative information that can support retrospective intrafraction motion assessment during bone metastases stereotactic body radiotherapy. This workflow may help identify fractions requiring further review and support offline quality assurance, although prospective validation is required before clinical implementation.

## Highlights

• Triggered images retrospectively quantified motion in bone metastases radiotherapy.
• The workflow was applied to 8717 images from 183 fractions in 49 patients.
• Clinically relevant motion (>2 mm) detected in 5.7% of cases.
• Imaging angle affected displacement (*p* < 0.01); one center showed a 0.5 mm offset.
• The workflow may support offline quality assurance for bone metastases radiotherapy.

## Graphical abstract

**Figure:**
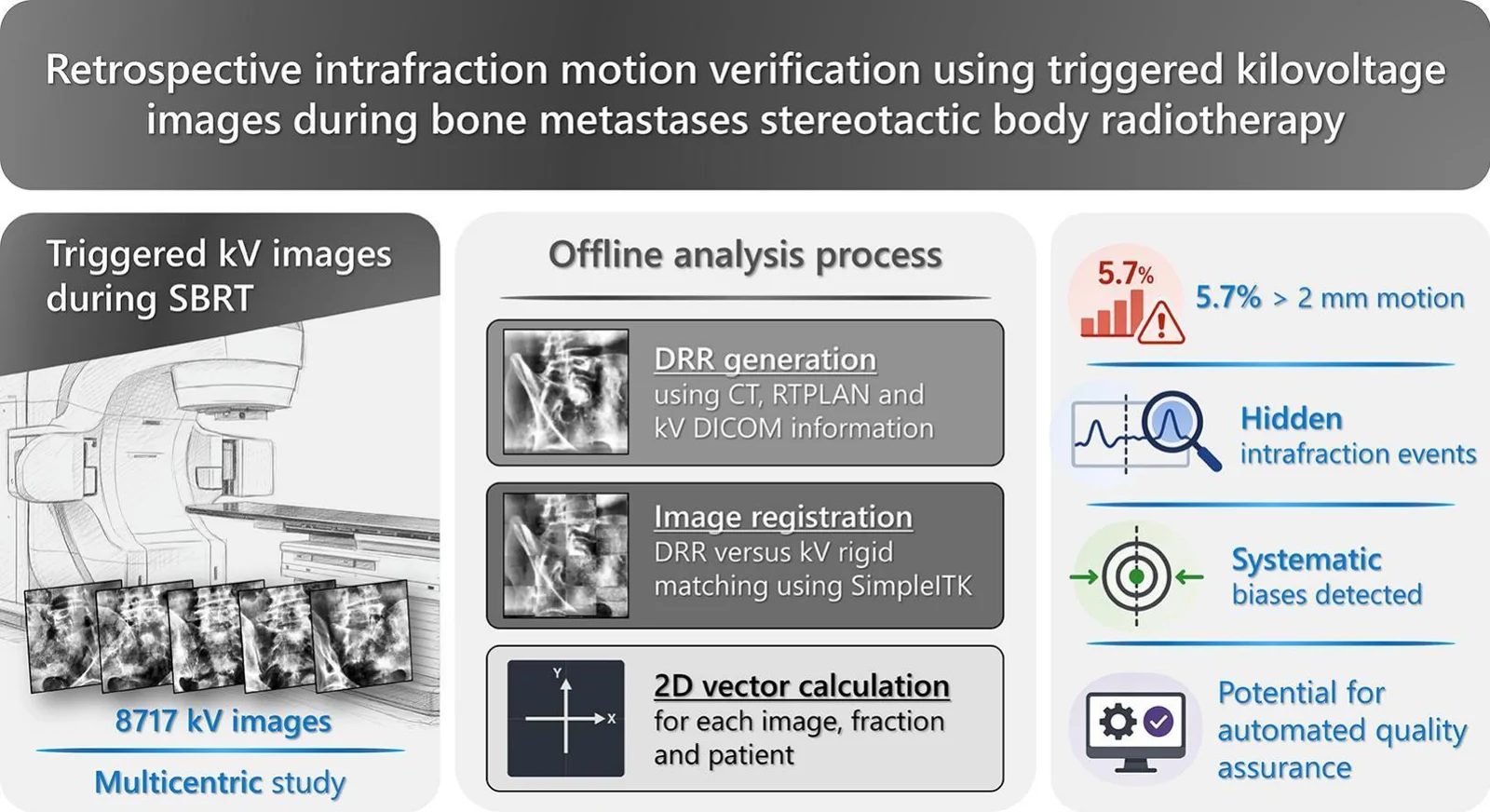

## 1 Introduction

Stereotactic body radiotherapy (SBRT) is increasingly used for patients with bone metastases, allowing delivery of high radiation doses with steep dose gradients while limiting exposure to critical structures, particularly the spinal cord [1], [2], [3]. Because of the high dose per fraction and small planning target volume (PTV) margins, accurate patient positioning and intrafraction motion management are essential. Even small anatomical displacements may compromise target coverage or increase the dose to nearby organs of interest [4], [5], [6].

Several strategies are available for intrafraction monitoring. Surface imaging provides continuous monitoring of external motion but does not necessarily reflect internal anatomical motion, especially for deep bony structures such as the vertebral column [7], [8], [9], [10]. Image-guided radiotherapy (IGRT) solutions based on kilovoltage (kV) imaging can provide direct internal verification, but acquisition frequency is limited and image interpretation may require manual focus on the treated vertebral level or its vicinity [11]. Fiducial tracking may improve localization accuracy, but requires an invasive procedure that is often unsuitable for patients treated for bone metastases [12]. Robotic radiotherapy systems and magnetic resonance-guided linear accelerators provide advanced tracking or visualization capabilities, but remain limited to specialized platforms [13], [14], [15], [16], [17], [18], [19].

In contrast, triggered kV imaging is available on most conventional linear accelerators and can be acquired during treatment delivery [20], [21], [22], [23]. These images are typically acquired at predefined intervals or gantry angles and are mainly used for visual verification. However, interpretation can be challenging because of limited image contrast, frequent acquisition, and the need to simultaneously monitor both the patient and treatment system [24], [25], [26]. As a result, the quantitative information contained in triggered imaging remains largely underexploited.

Previous studies have highlighted the value of intrafraction kV imaging during spine or bone metastases SBRT. Remmerts de Vries et al. analyzed intrafraction motion during spine SBRT using high-frequency kV imaging and a dedicated research tool [21], while Hadj Henni et al. investigated per-irradiation monitoring in stereotactic treatment of spinal and non-spinal bone metastases, mainly relying on visual image assessment [26]. These studies support the clinical relevance of bone position monitoring. However, systematic retrospective exploitation of routinely acquired triggered images using an open, reproducible, and center-adaptable workflow remains limited.

The aim of this study was to develop and evaluate a retrospective workflow for intrafraction motion verification using triggered kV images during bone metastases SBRT. The pipeline extracts triggered images and planning data, generates digitally reconstructed radiographs (DRRs) from the planning computed tomography (CT), and performs focal rigid image registration. We aimed to support intrafraction bone motion assessment, provide a workflow for retrospective quality assurance using routine data, and demonstrate that visual inspection does not fully exploit triggered imaging information.

## 2 Materials and methods

### 2.1 Study cohort

Between 2022 and 2025, 49 patients treated with SBRT for bone metastases were included from two radiotherapy centers, referred to as Center A and Center B. Treatments were delivered on Varian linear accelerators (Varian, a Siemens Healthineers Company, Palo Alto, CA, USA), including a TrueBeam STx system with Brainlab ExacTrac Dynamic (Brainlab, Munich, Germany) surface monitoring at Center A and a TrueBeam system with AlignRT (Vision RT Ltd., London, United Kingdom) surface monitoring at Center B. A total of 183 treatment fractions was analyzed. According to institutional practice and clinical indication, treatments were delivered in one, three, or five fractions using isotropic planning target volume (PTV) margins of 2 mm and 3 mm in Centers A and B, respectively. Patients were immobilized according to treatment site and local practice, using site-adapted devices such as thermoplastic masks, vacuum cushions, or body immobilization systems.

This retrospective observational study was conducted using previously acquired routine-care imaging and treatment-planning data. According to national regulations and local institutional procedures applicable to research not involving human participants, formal approval by an ethics committee was not required. This applied to both participating centers. All patients provided informed consent for the use of their anonymized data for research purposes. Clinical characteristics of the study cohort are summarized in Table 1.

**Table 1:** Descriptive patient characteristics and radiotherapy parameters. For the first three rows, percentages in the Center A and Center B columns refer to the proportion of the total cohort, total number of fractions, or total number of triggered kilovoltage images, respectively. For fractionation and number of simultaneously treated volumes, percentages were calculated relative to the number of patients or treatment courses in each group. For anatomical sites, percentages were calculated relative to the total number of treated volumes in each group. Triggered kV acquisition parameters are reported as mean (range). Tube voltage, tube current, and exposure time were summarized across individual triggered images, whereas total triggered-imaging duration was summarized across treatment fractions.

	Total (%)	Center A (%)	Center B (%)
Number of patients	49	32 (65.3)	17 (34.7)
Number of fractions	183	160 (87.4)	23 (12.6)
Number of triggered images	8717	6177 (70.9)	2540 (29.1)
Fractionation			
1 fraction	14 (28.6)	0 (0.0)	14 (82.3)
3 fractions	3 (6.1)	0 (0.0)	3 (17.7)
5 fractions	32 (65.3)	32 (100)	0 (0.0)
Treated volumes by anatomical site			
Cervical	7 (11.5)	5 (10.6)	2 (14.3)
Thoracic	26 (42.6)	20 (42.6)	6 (42.9)
Lumbar	19 (31.1)	15 (31.9)	4 (28.6)
Sacral	4 (6.6)	3 (6.4)	1 (7.1)
Other bone	5 (8.2)	4 (8.5)	1 (7.1)
Number of simultaneously treated volumes			
1	40 (81.6)	25 (78.1)	15 (88.2)
2	8 (16.3)	6 (18.8)	2 (11.8)
3	1 (2.0)	1 (3.1)	0 (0.0)
Triggered kV acquisition parameters, mean (range)			
Tube voltage [kVp]	101.8 (65–140)	102.4 (70–140)	100.3 (65–135)
Tube current [mA]	131.8 (70–235)	130.1 (77–234)	135.7 (70–235)
Exposure time [ms]	54.9 (25–420)	55.2 (50–83)	52.0 (25–420)
Total triggered-imaging duration per fraction [s]	162.7 (59.5–544.2)	138.3 (88.4–166.8)	278.0 (59.5–544.2)

### 2.2 Triggered kV image acquisition, data extraction, DRR generation and preprocessing

During treatment delivery, triggered kV images were automatically acquired using the accelerator on-board imaging systems according to the imaging protocols configured on each treatment unit. For this retrospective study, all triggered kV images associated with the analyzed fractions were retrieved in Digital Imaging and COmmunications in Medicine (DICOM) format, together with the corresponding planning CT, structure set, and treatment plan files. Triggered kV acquisition parameters were not prospectively standardized across centers: images were acquired during treatment delivery along with the MV beam on every 10° to 20°, depending on the center and treatment plan, using anatomical presets selected according to local practice and treatment site.

For each triggered kV image, a corresponding DRR was generated from the planning CT using the isocenter position and gantry angle extracted from the DICOM header. DRR generation was performed with DiffDRR 0.5.1, which is based on a differentiable implementation of Siddon-based ray tracing [27], [28]. A bone attenuation multiplier of 3 was empirically selected to enhance bony contrast and was kept constant for all analyses.

Before registration, triggered kV images and DRRs underwent preprocessing to improve image comparability. Histogram equalization was applied to normalize global intensity distributions, followed by contrast-limited adaptive histogram equalization to enhance local contrast. A Gaussian smoothing filter was applied to triggered kV images to reduce high-frequency noise. Finally, both triggered kV images and DRRs were cropped to a 15 cm × 15 cm region of interest centered on the treatment isocenter, focusing the analysis on the treated bony region while limiting the influence of surrounding anatomy. Image processing was implemented in Python using SimpleITK, OpenCV, and scikit-image [29], [30], [31], [32], [33].

### 2.3 Image registration and motion analysis

Rigid image registration was performed between each triggered kV image and its corresponding DRR using SimpleITK. Registration was limited to in-plane translations to provide a robust two-dimensional displacement estimate across projection angles and centers. Image similarity was evaluated using the Mean Squares metric, which was retained after preliminary testing because it provided the most stable registrations in this dataset after intensity normalization and contrast-enhancement preprocessing.

Registration was performed using a multi-resolution pyramid strategy and Regular Step Gradient Descent optimization. Optimizer parameters were empirically selected during preliminary testing and kept constant for all analyses. The resulting transformation parameters were used to estimate translational displacement in the X-axis direction, corresponding to left-right or anterior-posterior displacement depending on gantry angle, and in the Y-axis direction, corresponding to superior-inferior displacement. The displacement vector norm in the XY plane was also calculated.

The analysis focused on detecting displacement events exceeding a conservative threshold of 2 mm. Sustained events were defined as displacements greater than 2 mm on three consecutive images, in order to reduce the influence of isolated registration abnormalities. Given the 3–5 s interval between triggered images, these events corresponded to approximately 9–15 s of sustained displacement. Descriptive statistics were computed for X-displacement, Y-displacement, and displacement vector magnitude. Exploratory analyses included Mood's median test to assess differences in displacement medians across 20° gantry-angle bins separately for each center, and linear regression to evaluate trends in displacement vector magnitude according to image order within arcs, used as an approximate surrogate of acquisition sequence.

Computation times included preprocessing and registration but excluded DRR generation and file input/output. Analyses ran on a clinical workstation with a 20-core 3.19 GHz CPU and 64 GB RAM, without GPU acceleration. The image-level workflow, documentation, and pinned dependencies are publicly available on GitHub; version 1.0.0 is archived on Zenodo [34].

### 2.4 System evaluation

The performance of the semi-automated motion detection workflow was evaluated in two complementary steps.

First, a whole-body phantom (Kyoto Kagaku, Kyoto, Japan) including rib, humerus, and vertebral structures was imaged at the treatment isocenter and after known physical translations of 10 mm in all directions. This experiment provided a controlled geometric test with actual phantom displacements. Agreement between known and measured X-displacements was assessed using Bland-Altman analysis after checking the normality of the differences, with bias defined as the mean difference and limits of agreement as ±1.96 standard deviations [35]. Root mean square error (RMSE) was calculated as a complementary accuracy metric. For the Y-direction, where the imposed displacement was fixed, agreement was summarized using error-based metrics.

Second, a subset of triggered kV patient datasets was selected for each vertebral level and visually reviewed by medical physicists experienced in image-guided radiotherapy to exclude datasets with apparent intrafraction displacement, poor image quality, or inconsistent anatomical alignment across consecutive images. Simulated isocenter shifts of 5 mm and 10 mm were then introduced in all directions before generating the corresponding DRRs. This second step was designed to challenge the registration workflow under deliberate misalignment conditions while preserving realistic clinical image characteristics, including patient-specific contrast, noise, and anatomical superposition.

## 3 Results

The registration workflow produced color fusion and checkerboard overlays for visual assessment of registration quality, with representative outputs provided in Supplementary Fig. S1.

In the centered phantom configuration, the mean displacement vector norm was 0.4 ± 0.26 mm, with 97.0% of measurements showing a displacement vector magnitude below 1 mm. After physical phantom displacement, the workflow detected a mean Y-direction displacement of 10.04 ± 0.67 mm. Bland-Altman analysis of X-direction displacement showed good agreement between known and measured displacements, with RMSE values of 0.7 mm in both the X- and Y-directions (Supplementary Fig. S2). In patient datasets with simulated shifts, aberrant registrations were identified in 0.3% of 5-mm displacement tests and 5.8% of 10-mm displacement tests, with most failures occurring for shifts along the vertebral axis. Detailed results for each test condition are provided in Table 2.

**Table 2:** Phantom and patient-based system evaluation of the registration workflow under different validation conditions, including deliberate phantom shifts of 10 mm and simulated shifts of 5 mm and 10 mm applied to patient images. Residual errors were calculated as the differences between measured and expected displacement components. The residual vector error was defined as the Euclidean norm of these differences. Results are reported as mean ± standard deviation in the X-direction, Y-direction, and displacement vector magnitude in the XY plane, together with the percentage of measurements with a displacement vector magnitude <1 mm.

Test condition	Number of images	Mean residual error in X-direction [mm]	Mean residual error in Y-direction [mm]	Mean residual vector error [mm]	Residual vector error < 1 mm [%]
Phantom centered	134	0.07 (± 0.41)	0.02 (± 0.22)	0.4 (± 0.26)	97%
Phantom (10 mm shifts)	147	0.04 (± 0.76)	0.0 (± 0.66)	0.84 (± 0.56)	68%
Patients (5 mm shifts)	291	0.0 (± 0.18)	0.0 (± 0.11)	0.16 (± 0.14)	99.7%
Patients (10 mm shifts)	291	−0.11 (± 3.2)	−1.43 (± 6.47)	1.88 (± 7.12)	94.2%

**Fig. 1:**
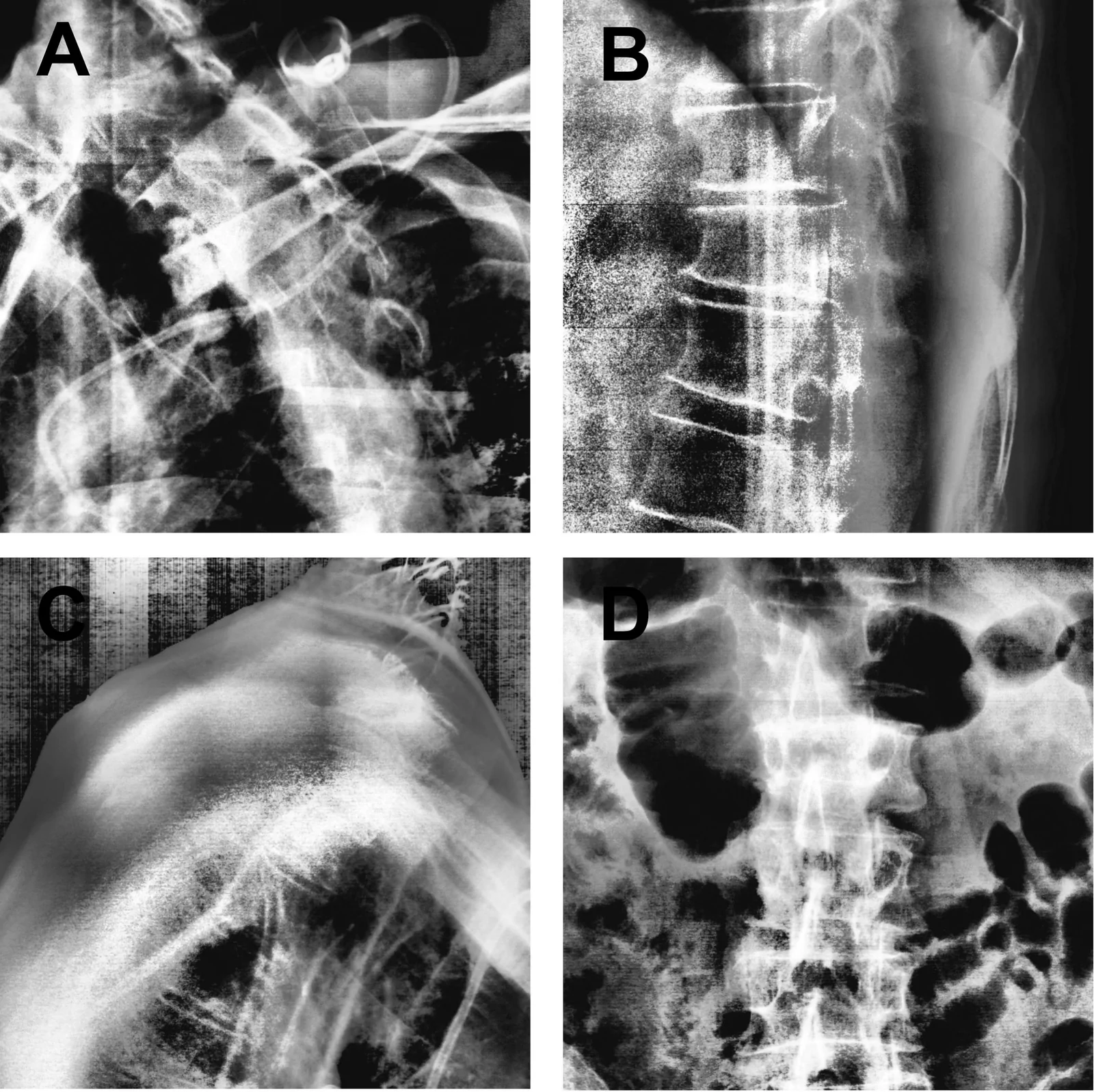
Examples of triggered kV images that resulted in aberrant registrations and were excluded from the analysis. Different causes of registration failure are illustrated: presence of accessories in the imaging field (A), poor image contrast (B), arms overlapping the spine (C) and abdominal gas affecting image similarity (D).

In the full cohort, 8717 triggered kV images were analyzed. Correct registration was obtained for 7105 images (81.5%), whereas 1612 images (18.5%) were classified as aberrant registrations, including isolated discrepancies (2.1%) and systematic errors (16.4%). Isolated discrepancies corresponded to occasional failed registrations within otherwise exploitable image series, whereas systematic errors corresponded to image series or fractions considered unreliable because of persistent image-quality or workflow-related issues. Aberrant cases were visually attributed to accessories or artifacts visible on triggered kV images, poor image contrast related to suboptimal acquisition parameters, and overlapping anatomical structures, such as arms or gas (Fig. 1), and were excluded from subsequent motion analysis. Two aberrant cases presented highly pixelized DRRs, attributed to planning CT acquisitions performed with 2-mm and 3-mm slice thickness instead of 1 mm.

After exclusion of aberrant registrations, the mean displacement vector norm was 1.2 ± 0.8 mm in the total population, with similar values in Center A and Center B (1.2 ± 0.8 mm and 1.1 ± 0.7 mm, respectively). Overall, 3398 of 7105 images (47.8%) showed a displacement vector below 1 mm. Sustained displacement events were observed in 405 of 7105 images (5.7%). At the fraction level, 46 of 159 fractions (28.9%) showed at least one sustained event, including 39 of 136 fractions (28.7%) in Center A and 7 of 23 fractions (30.4%) in Center B (Table 3). Overall, 85% of measurements showed displacements ≤2 mm and 98% showed displacements ≤3 mm. Observed events included sustained offsets over consecutive images and more isolated transient excursions.

**Table 3:** Results of displacement measurements in the patient cohort after exclusion of aberrant registrations, reported for each center and for the total population. Continuous variables are reported as mean ± standard deviation. Proportions are reported as number (%), using the number of images as denominator for image-level results and the number of fractions as denominator for fraction-level results. The displacement vector magnitude corresponds to the magnitude of the two-dimensional displacement in the image plane. Sustained displacement events were defined as displacements greater than 2 mm on three consecutive images.

Condition	Total population	Center A	Center B
Number of images	7105	5282	1823
Number of fractions	159	136	23
Number of patients	45	28	17
Mean displacement in X-direction [mm]	−0.1 ± 1.0	−0.2 ± 1.0	0.0 ± 0.9
Mean displacement in Y-direction [mm]	0.1 ± 1.0	0.0 ± 1.0	0.5 ± 0.8
Mean displacement vector magnitude [mm]	1.2 ± 0.8	1.2 ± 0.8	1.1 ± 0.7
Images with displacement vector magnitude <1 mm (%)	3398 (47.8)	2377 (45.0)	1021 (56.0)
Images belonging to sustained events (%)	405 (5.7)	320 (6.1)	85 (4.7)
Fractions with ≥1 sustained event (%)	46 (28.9)	39 (28.7)	7 (30.4)

In the five-fraction treatments from Center A, mean displacement vector magnitudes remained broadly stable across fractions, with values of 1.2, 1.4, 1.2, 1.2, and 1.2 mm from fractions 1 to 5, respectively. The proportion of fractions with at least one sustained displacement event did not show a clear evolution over the treatment course, with values of 32.1%, 32.1%, 25.0%, 26.9%, and 26.9%. Analysis according to image order within arcs did not reveal progressive intrafraction drift, with mean displacement vector magnitudes of 1.2 mm in the early, middle, and late thirds of the image sequence, respectively, and no significant linear trend across image order (*p* = 0.80).

**Fig. 2:**
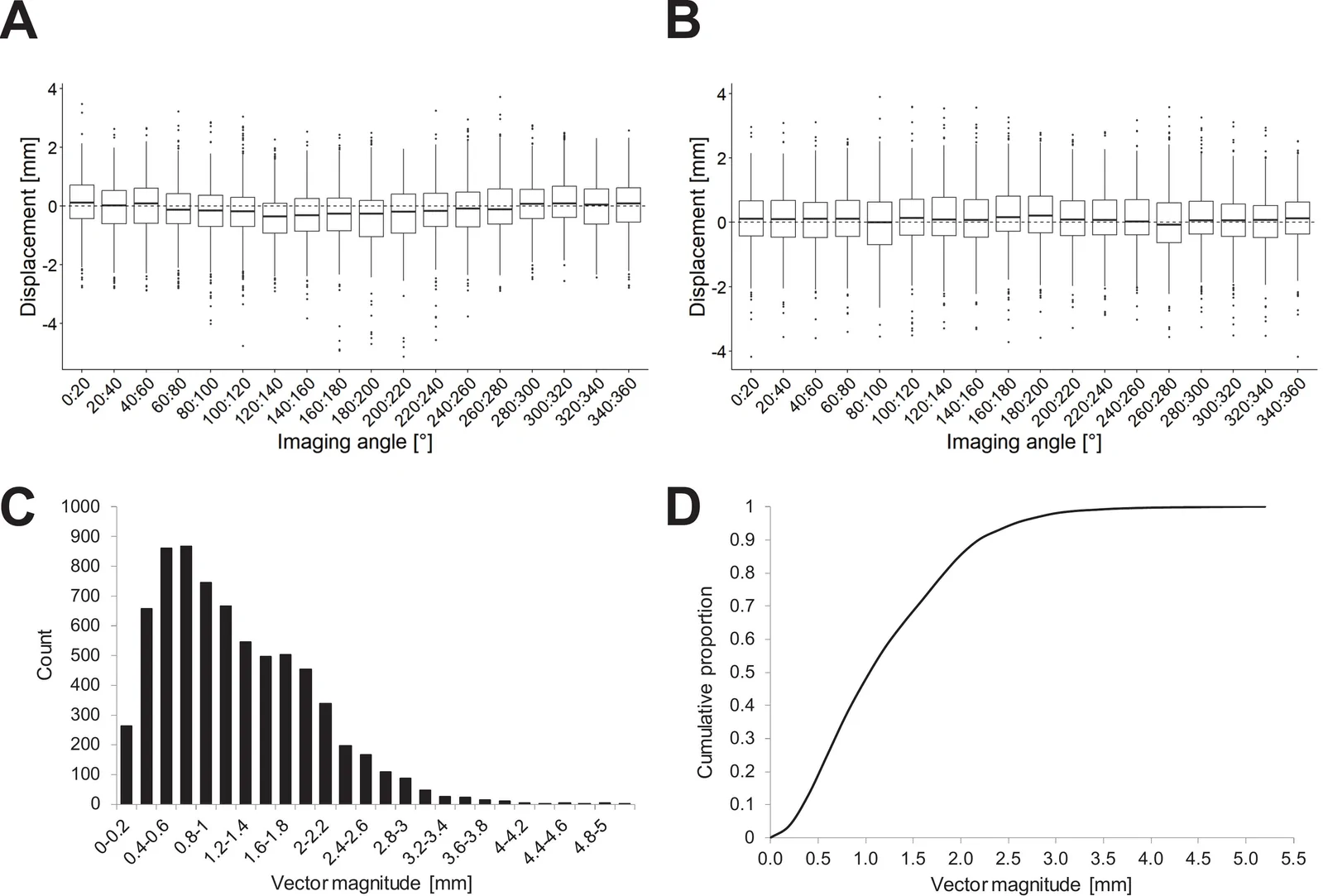
Distribution and characterization of intrafraction displacement measurements after exclusion of aberrant registrations for the entire cohort. (A) Box-and-whisker plots of displacement values along the X-direction (mm) as a function of kV imaging angle. (B) Box-and-whisker plots of displacement values along the Y-direction (mm) as a function of kV imaging angle. (C) Distribution of displacement vector magnitudes (mm) in the XY plane for the full cohort. (D) Cumulative distribution of displacement vector magnitudes, expressed as the proportion of measurements below a given threshold.

Angular analysis showed that most displacement measurements remained centered around 0 mm, although distribution tails were observed in both displacement components and in the displacement vector magnitude (Fig. 2). Analysis using 20° gantry-angle bins showed a significant dependence of median X-displacement on gantry angle in both centers (Mood's median test: *p* < 0.01 for both centers), whereas no robust median dependence was observed for Y-displacement or displacement vector magnitude. Center-specific angular distributions are provided in Supplementary Figs. S3 and S4. At Center B, a consistent offset of approximately 0.5 mm in the Y-direction across angles was also observed (Supplementary Fig. S3A,B).

The mean computation time per image preprocessing and registration was 1699 ms (± 504 ms).

## 4 Discussion

In this study, we developed and evaluated a retrospective workflow for intrafraction motion verification using triggered kV images acquired during bone metastases SBRT on conventional linear accelerators. Applied to 8717 images from 183 fractions in 49 patients, the workflow successfully registered 81.5% of the images. The mean displacement vector magnitude was 1.2 ± 0.8 mm, while sustained displacements exceeding 2 mm were detected in 5.7% of analyzable images and 28.9% of fractions. These findings demonstrate the feasibility of using routinely acquired triggered imaging for large-scale retrospective motion assessment and offline quality assurance without additional imaging or dedicated hardware.

These findings are consistent with previous reports supporting the value of intrafraction kV imaging for position verification during spine and bone metastases SBRT [21], [26]. A particularly comparable workflow was described by Fan et al., who registered triggered kV images acquired using the TrueBeam Intrafraction Motion Review system with planning CT-derived DRRs. Among 1371 images with valid registration results, 6.6% showed a displacement ≥2 mm in at least one in-plane direction [36]. This proportion is of the same order as the sustained displacement events observed in our cohort, although the definitions are not directly equivalent. Fan et al. applied a per-image, axis-specific threshold during fixed-field intensity modulated field, whereas we evaluated the two-dimensional vector magnitude during arc delivery and required three consecutive images to define a sustained event. Furthermore, their proprietary workflow was designed for near-real-time treatment guidance, whereas our open and center-adaptable workflow was developed for large-scale retrospective analysis and offline quality assurance.

Triggered imaging contains quantitative information that remains largely underexploited. In bone SBRT, high doses per fraction, steep dose gradients, and small PTV margins make geometric accuracy particularly important [1], [2], [3], [4], [5], [6], [21], [24], [25], [26]. The 2-mm threshold used in this study should therefore be interpreted as a conservative quality-assurance threshold rather than as direct evidence of PTV margin violation. Although most measured displacements were small, sustained events above the predefined quality-assurance threshold occurred in a subset of fractions, suggesting that triggered imaging may help identify fractions requiring retrospective review. The multicenter design further supports feasibility across different clinical environments, while the angular dependence of X-displacement and small systematic Y-direction offset in one center suggest that this approach may also reveal center- or system-specific biases, although such patterns may also be influenced by projection geometry and the limitations of the in-plane translation-only model.

Available intrafraction monitoring technologies may rely on external surrogates, intermittent imaging, invasive markers, manual interpretation, or specialized platforms [17]. In contrast, triggered kV imaging is available on many conventional linear accelerators and provides direct visualization of internal bony anatomy [21], [24], [25], [26]. Although this study remains a retrospective offline quality-assurance analysis rather than validation of a real-time monitoring system, the detection of sustained displacement events supports further development of automated image-based verification strategies for bone SBRT. Similar frameworks could also be explored for other indications using bony anatomy as a surrogate, provided acquisition protocols, registration settings, and quality-control procedures are adapted to the clinical objective [37], [38].

The proportion of aberrant registrations observed in this study represents an important limitation. These cases were systematically identifiable and mainly associated with poor image quality, anatomical overlap, artifacts, or workflow-related issues. However, interobserver variability was not formally assessed for this visual quality-control step. This rate should therefore not be interpreted as the expected false-trigger rate of a prospective online implementation, since the images and planning data were not acquired or optimized for automated registration. Rather, these findings support the need for automated quality-control strategies, potentially based on final similarity metric values, image-quality indicators, anatomical consistency across consecutive images, or outlier displacement patterns [39], [40], [41]. Prospective optimization of acquisition presets, improved DRR generation, and exclusion criteria for unreliable projections may also reduce aberrant registrations.

Several additional limitations should be acknowledged. First, the study was not powered to identify patient-level or lesion-specific predictors of intrafraction displacement, and future studies should account for within-patient correlation when investigating factors such as anatomical site, immobilization, fractionation, re-irradiation status, or patient-specific anatomy. Triggered kV acquisition parameters were also not prospectively harmonized across centers, which may have contributed to variability in image quality and registration performance. Second, registration was limited to in-plane translations and did not account for rotational or deformable variations. Third, although preprocessing improved image comparability, registration performance may still have been affected by residual contrast differences between triggered kV images and DRRs, artifacts, anatomical variations, or suboptimal imaging conditions. A dedicated sensitivity analysis of DRR generation parameters, image contrast, preprocessing choices, and acquisition settings was not performed and should be considered in future validation studies.

Validation combined controlled phantom experiments, simulated patient shifts under realistic imaging conditions, and multicenter clinical registrations. The first two components assessed technical accuracy, whereas the clinical analysis evaluated performance across heterogeneous anatomy, image quality, artifacts, and DRR/triggered-image mismatch.

Finally, the current CPU-based implementation is intended for offline analysis. Code optimization and hardware acceleration would be required before considering prospective real-time use [17], [42]. The impact of sustained displacement events on calculated dose/volume metrics was not evaluated; these events should therefore be interpreted as indicators requiring review rather than as direct evidence of target underdosage or organ of interest overdose. Future studies combining motion detection with dose recalculation or dose accumulation will be required to determine their clinical significance.

Despite these limitations, this work demonstrates the feasibility of large-scale retrospective analysis of triggered imaging acquired during bone SBRT. The proposed workflow may help identify fractions, imaging conditions, and workflow-related factors requiring further investigation, and may contribute to broader integration of quantitative intrafraction imaging into radiotherapy quality assurance.

## CRediT authorship contribution statement

**Quentin Techer:** Software, Investigation, Formal analysis, Data curation, Conceptualization. **Noé Grandgirard:** Writing – original draft, Software, Methodology, Formal analysis, Data curation, Conceptualization. **Saturnin Sandjong:** Visualization, Methodology, Investigation, Formal analysis, Conceptualization. **Vincent Marchesi:** Writing – review & editing, Validation, Supervision, Resources, Project administration. **Paul Retif:** Writing – original draft, Visualization, Validation, Supervision, Project administration, Investigation, Formal analysis, Data curation.

## Code availability

The source code and documentation for the image-level processing workflow are publicly available through GitHub. Version 1.0.0 is permanently archived on Zenodo [34]. Clinical DICOM datasets are not publicly available because they contain sensitive patient information and are subject to institutional and regulatory restrictions.

## Declaration of competing interest

The authors declare that they have no known competing financial interests or personal relationships that could have appeared to influence the work reported in this paper.
