# Supplementary material for: Retrospective intrafraction motion verification using triggered kilovoltage images during bone metastases stereotactic body radiotherapy
Source: Phys Imaging Radiat Oncol. 2026 Aug 3;40:101054. doi: 10.1016/j.phro.2026.101054 (PMC13495594; doi:10.1016/j.phro.2026.101054)
Supplement: Supplementary file 1 [file mmc1.pdf]

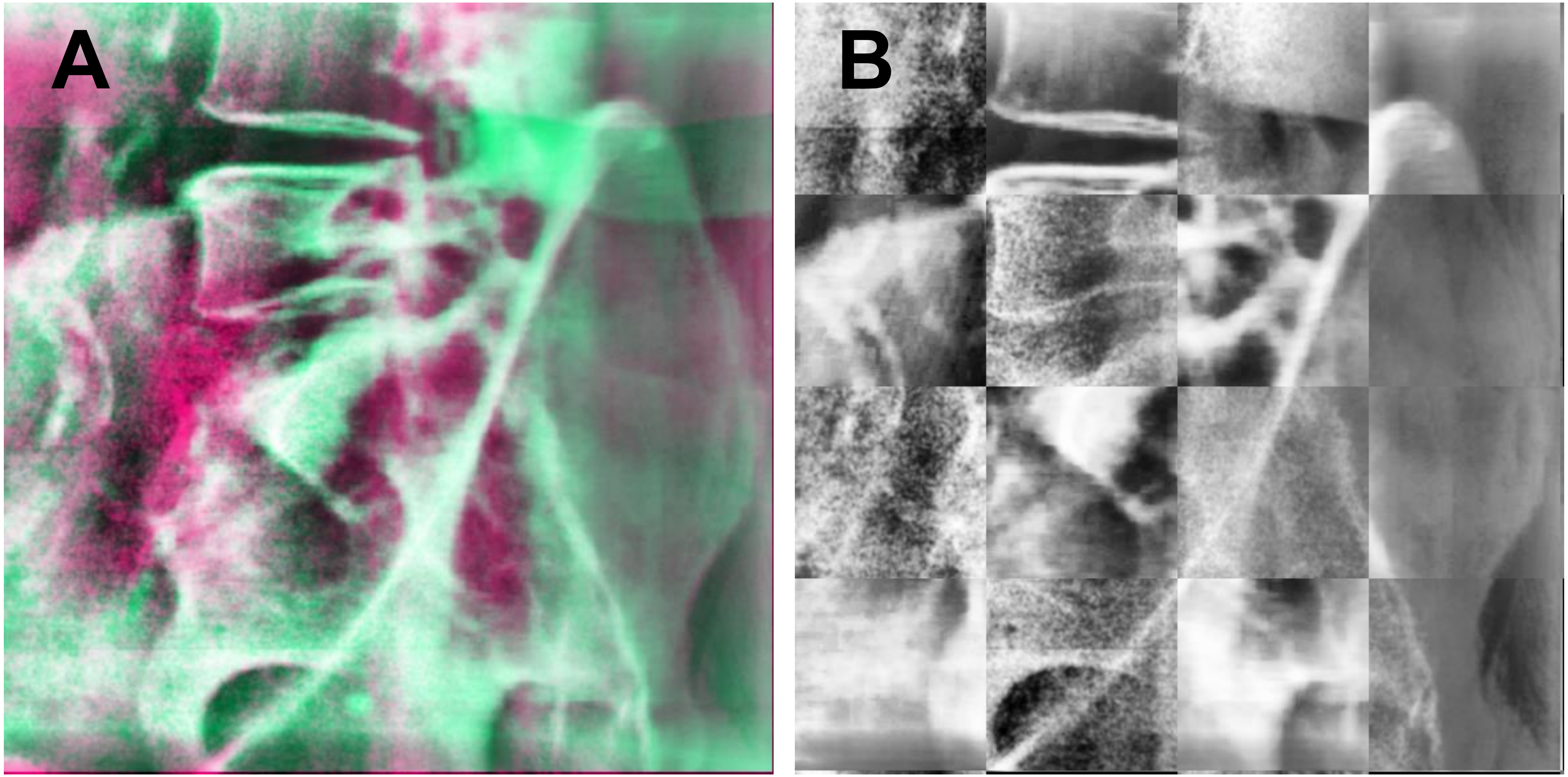

SUPPLEMENTARY FIGURE S1

Representative outputs of the image registration workflow. Overlay between pkV images and corresponding DRRs is shown using two visualization approaches: color fusion (A), where misalignments appear as color discrepancies, and checkerboard display (B), enabling local assessment of alignment between anatomical structures.

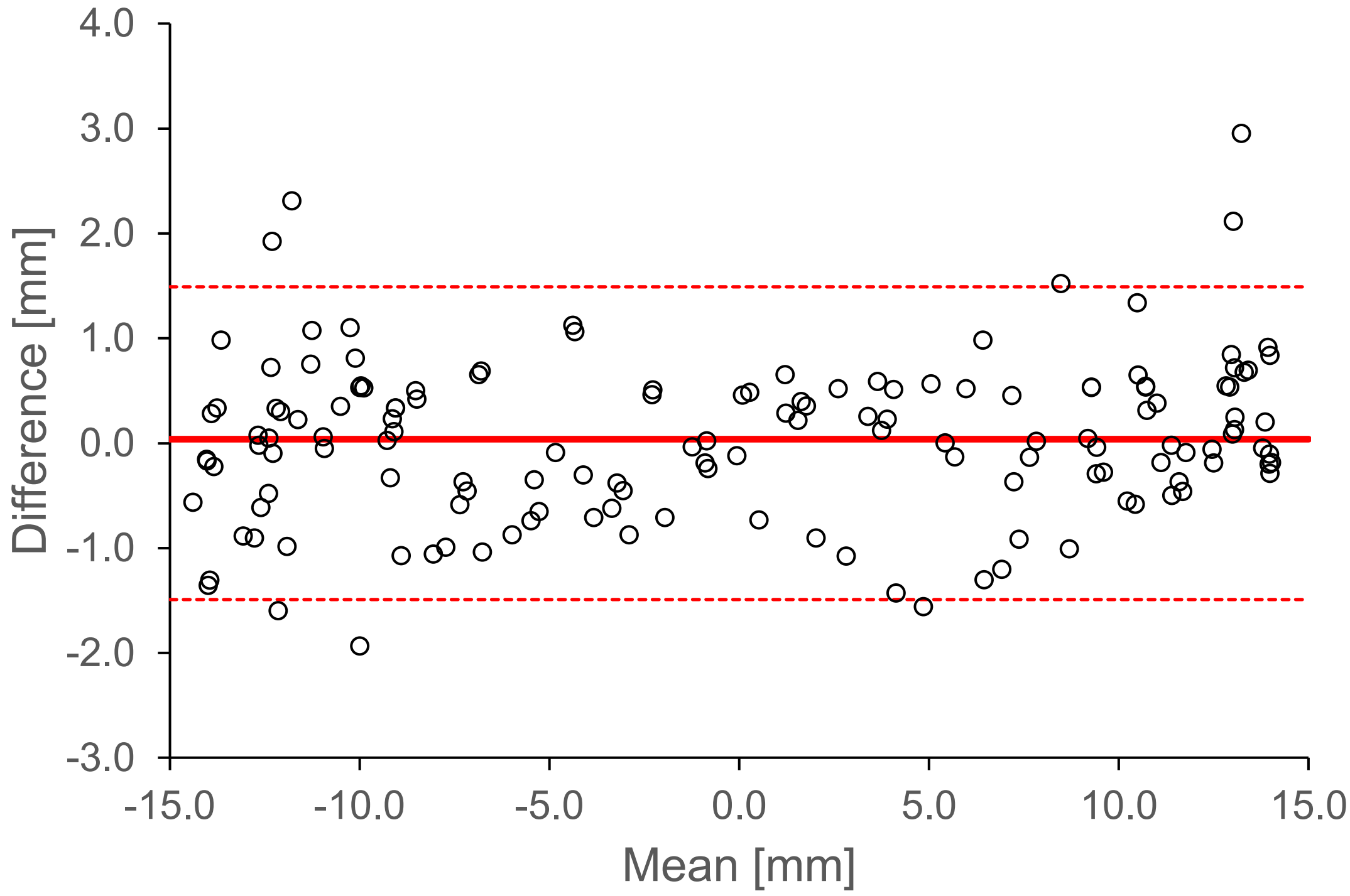

SUPPLEMENTARY FIGURE S2

Bland–Altman analysis of agreement between known and measured X-displacements in the phantom evaluation. The solid line represents the mean difference (bias), and the dashed lines represent the limits of agreement, defined as the mean difference ±1.96 standard deviations.

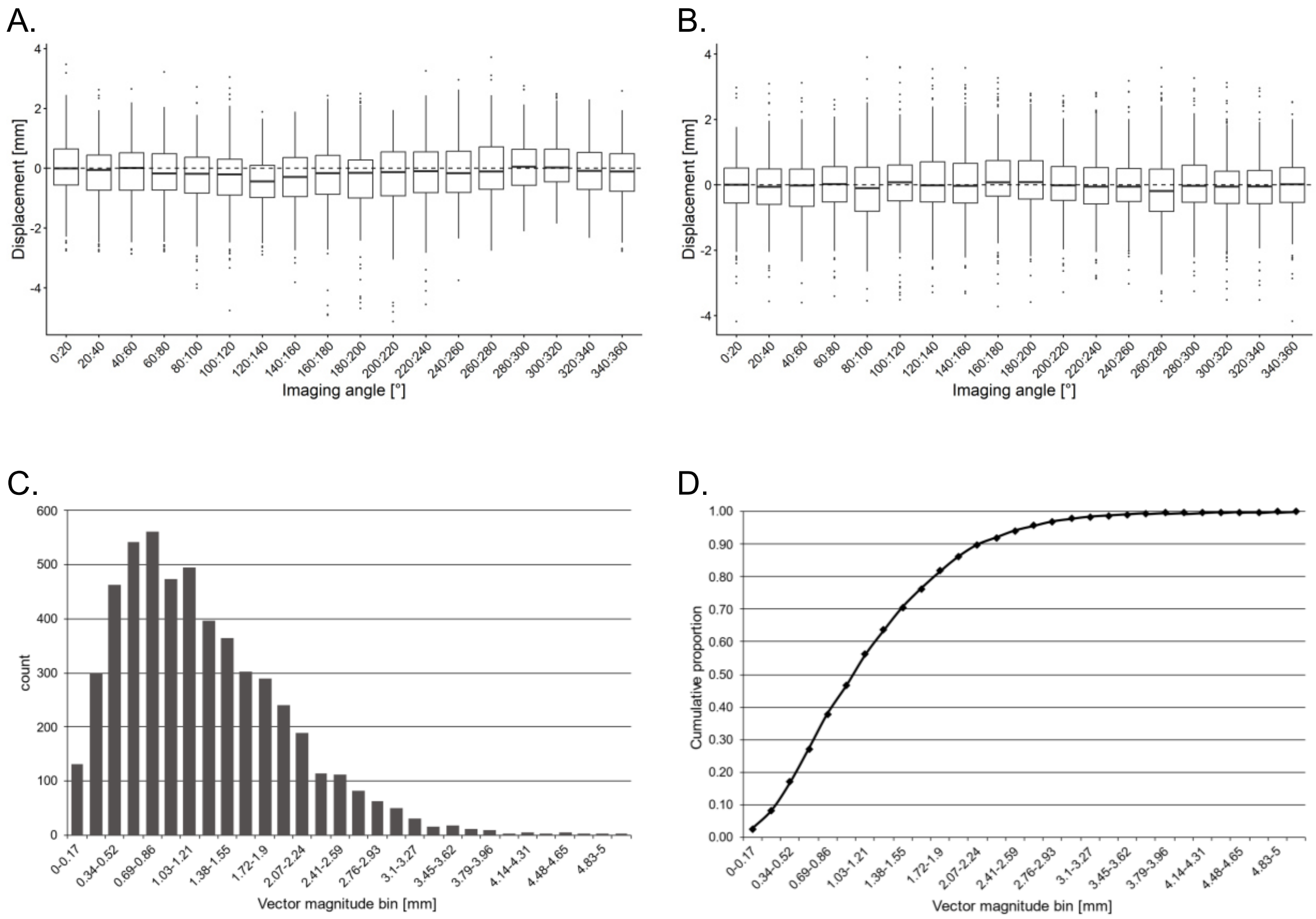

SUPPLEMENTARY FIGURE S3

Distribution and characterization of intrafraction displacement measurements after exclusion of aberrant registrations for the center A (A) Box-and-whisker plots of displacement values along the X-direction (mm) as a function of kV imaging angle. (B) Box-and-whisker plots of displacement values along the Y-direction (mm) as a function of kV imaging angle. (C) Distribution of displacement vector magnitudes (mm) in the XY plane for the full cohort. (D) Cumulative distribution of displacement vector magnitudes, expressed as the proportion of measurements below a given threshold.

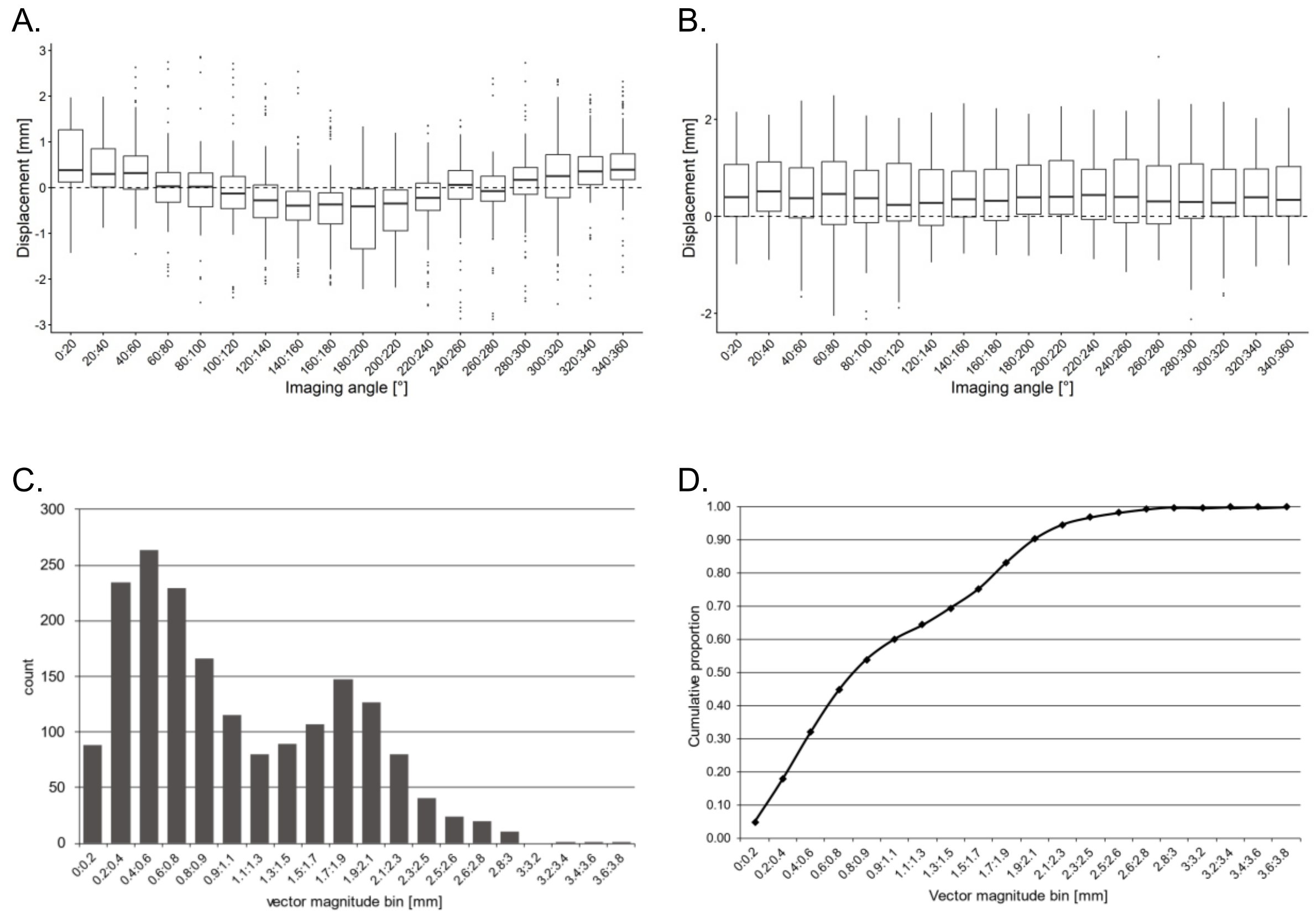

SUPPLEMENTARY FIGURE S4

Distribution and characterization of intrafraction displacement measurements after exclusion of aberrant registrations for the center B (A) Box-and-whisker plots of displacement values along the X-direction (mm) as a function of kV imaging angle. (B) Box-and-whisker plots of displacement values along the Y-direction (mm) as a function of kV imaging angle. (C) Distribution of displacement vector magnitudes (mm) in the XY plane for the full cohort. (D) Cumulative distribution of displacement vector magnitudes, expressed as the proportion of measurements below a given threshold.
